# Applying genomics in assisted migration under climate change: Framework, empirical applications, and case studies

**DOI:** 10.1111/eva.13335

**Published:** 2021-12-26

**Authors:** Zhongqi Chen, Lukas Grossfurthner, Janet L. Loxterman, Jonathan Masingale, Bryce A. Richardson, Travis Seaborn, Brandy Smith, Lisette P. Waits, Shawn R. Narum

**Affiliations:** ^1^ Aquaculture Research Institute University of Idaho Hagerman Idaho USA; ^2^ Bioinformatics and Computational Biology Graduate Program University of Idaho Hagerman Idaho USA; ^3^ Department of Biological Sciences Idaho State University Pocatello Idaho USA; ^4^ Rocky Mountain Research Station USDA Forest Service Moscow Idaho USA; ^5^ Department of Fish and Wildlife Resources University of Idaho Moscow Idaho USA; ^6^ Columbia River Inter‐Tribal Fish Commission Hagerman Idaho USA

**Keywords:** adaptation, assisted gene flow, assisted migration, climate change, conservation, genomics

## Abstract

The rate of global climate change is projected to outpace the ability of many natural populations and species to adapt. Assisted migration (AM), which is defined as the managed movement of climate‐adapted individuals within or outside the species ranges, is a conservation option to improve species' adaptive capacity and facilitate persistence. Although conservation biologists have long been using genetic tools to increase or maintain diversity of natural populations, genomic techniques could add extra benefit in AM that include selectively neutral and adaptive regions of the genome. In this review, we first propose a framework along with detailed procedures to aid collaboration among scientists, agencies, and local and regional managers during the decision‐making process of genomics‐guided AM. We then summarize the genomic approaches for applying AM, followed by a literature search of existing incorporation of genomics in AM across taxa. Our literature search initially identified 729 publications, but after filtering returned only 50 empirical studies that were either directly applied or considered genomics in AM related to climate change across taxa of plants, terrestrial animals, and aquatic animals; 42 studies were in plants. This demonstrated limited application of genomic methods in AM in organisms other than plants, so we provide further case studies as two examples to demonstrate the negative impact of climate change on non‐model species and how genomics could be applied in AM. With the rapidly developing sequencing technology and accumulating genomic data, we expect to see more successful applications of genomics in AM, and more broadly, in the conservation of biodiversity.

## 1 INTRODUCTION

The current rapid rate of climate change and increased variability are decoupling local populations from the climate conditions to which they adapted in the past (Ainsworth et al., 2016; Jump & Penuelas, 2005; Vázquez et al., 2017). The ability of these populations to respond and adapt to new environmental conditions will directly impact ecosystem processes and services at a local and global scale, making some ecosystems vulnerable to transformation (Seastedt et al., 2008). While some populations may tolerate these rapidly changing conditions through standing genetic variation (Barrett & Schluter, 2008; Brennan et al., 2019; Hancock et al., 2011), phenotypic plasticity (Gárate‐Escamilla et al., 2019; Reed et al., 2011; Richardson et al., 2017), epigenetic processes (Daxinger & Whitelaw, 2012; Weigel & Colot, 2012), evolutionary rescue through adaptation to new conditions (Anderson et al., 2012; Gonzalez & Bell, 2013), or migration to locations within their climatic niches based on predictive niche models (Loarie et al., 2009), others will not be able to overcome maladaptation to changing local conditions and will decline in abundance and/or be extirpated. This urgency has created a need to evaluate appropriate human interventions such as the translocation of individuals with climate‐adapted genes and heritable phenotypes (Bay et al., 2018; Butt et al., 2020; Gaitán‐Espitia & Hobday, 2021; Vitt et al., 2010; Wilkening et al., 2015).

**FIGURE 1:**
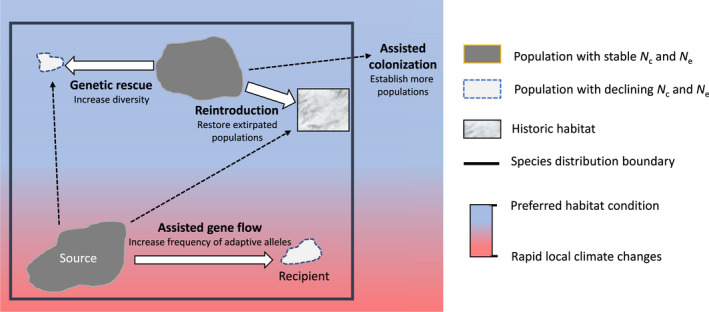
Conceptual diagram of assisted migration and other conservation management practices. The assisted migration of individuals with adapted alleles in response to climate change includes assisted gene flow within the historical species range and assisted colonization, which establishes populations outside of the historical range to new areas that have become or are projected to become suitable. Individuals can also be moved for other management reasons unrelated to climate change including reintroduction to extirpated regions and genetic rescue to increase genetic diversity in isolated populations that are experiencing negative fitness consequences such as inbreeding depression

Assisted migration (AM) is a conservation option in response to observed or anticipated climate change by intentional anthropogenic movement of climate‐adapted individuals within or outside the species ranges (McLachlan et al., 2007; Ste‐Marie et al., 2011; Vitt et al., 2010). Approaches for AM include movement of resilient individuals within the species range to areas that have been hard‐hit by changing climate, known as assisted gene flow, or movement of individuals outside of current ranges to new areas that have become or are projected to become suitable, known as assisted colonization. Assisted gene flow is sometimes referred to as a form of assisted evolution as it could accelerate adaptation. In this paper, we use AM as an overarching term and distinguish subcategories (i.e., assisted gene flow and assisted colonization). However, these terms are often used interchangeably in many other publications to refer to managed movement of individuals in response to environmental changes (e.g., Aitken & Whitlock, 2013; Hoegh‐Guldberg et al., 2008). Considering that AM focuses explicitly on movement of individuals to assist in climate change response, applications of AM have a distinct purpose of introducing adaptive genetic diversity to recipient populations in contrast to other conservation translocations such as genetic rescue that is intended to reduce genetic load in small, inbred populations (Hoffmann et al., 2021; Tallmon et al., 2004; Whiteley et al., 2015) (Figure 1). However, climate change may cause reduction in population size and additional complicated scenarios regarding genetic variation, which suggests that management options are not mutually exclusive and may need to integrate various strategies of genetic mixing to achieve intended conservation outcomes (Hoffmann et al., 2021).

Although there are scientific, legal, ethical and social controversies surrounding AM (Table 1; Filbee‐Dexter & Smajdor, 2019; McLachlan et al., 2007; Schwartz et al., 2012; Seddon, 2010; Vitt et al., 2010), many assert that the benefits of AM outweigh the potential costs (Hällfors et al., 2017; Minteer & Collins, 2010; Sáenz‐Romero et al., 2021). For species with specific habitat needs or those restricted by physical barriers or fragmentation, climate change may result in extinction of critical populations, making AM the best available strategy in certain circumstances (Thomas, 2011). Effective application of predictive climatic niche and genetic models, combined with knowledge of life history, could provide an opportunity for resource managers to utilize AM to mitigate some of the effects of climate change on biodiversity. However, candidate species should be evaluated individually, as successful implementation will require a balance between creating a self‐sustaining population with maintaining ecosystem function and preventing ecological or economic harm in the recipient region (Hällfors et al., 2017; Pérez et al., 2012; Richardson et al., 2009). For example, research has suggested that AM may be more appropriate for certain taxonomic groups, such as plants, as opposed to aquatic species, due to risks of invasions when introduced outside of the natural geographical range (Mueller & Hellmann, 2008). However, there may be many scenarios where AM could be applied in aquatic organisms and terrestrial animals to avoid extirpation of endemic populations by increasing adaptive capacity in a changing climate.

**TABLE 1:** Potential impacts of applying assisted migration as a conservation practice under climate change

Potential impacts of assisted migration	Reference example
Benefits	
Species persistence	Willis et al. (2009)
Ecosystem stability	Riegl et al. (2011)
Promotes climate adaptation	Gray et al. (2011)
Increases population size	Backus and Baskett (2020)
Risks	
Introduction of potentially invasive species	Ricciardi and Simberloff (2009)
Loss of genetic diversity	Kekkonen and Brommer (2015)
Introduction of maladaptive alleles	Wadgymar and Weis (2017)
Disrupts biotic interactions	Bucharova (2017)
Introduction of disease	Simler et al. (2019)

Note

A single example is given for each item, but text includes several more citations.

Successful application of AM faces many biological challenges. Researchers have suggested multiple strategies to increase success of AM, including choosing multiple source stocks or individuals with higher heterozygosity to maximize adaptive potential (Broadhurst et al., 2008; Bucharova et al., 2019; Hoffmann et al., 2021; Prober et al., 2015; Scott et al., 2020) or genetically matching stocks to the local conditions or projected local conditions (Rice & Emery, 2003; Sgrò et al., 2011). Multiple review articles have suggested that the application of genomic techniques that survey large portions of the genome, including both neutral and adaptive regions, could be useful and important in planning for AM and monitoring the success of AM (Aitken & Whitlock, 2013; Allendorf et al., 2010; Dumroese et al., 2015; Funk et al., 2012; Kelly & Phillips, 2019). For example, an increasing number of studies in a wide array of taxa are documenting standing allelic variation within a species that is associated with increased thermal tolerance (Hancock et al., 2011; Jackson et al., 1998; Narum et al., 2013; Pespeni et al., 2013).

The goal of this paper was to explore the current and future contributions of genetic and genomic methods to inform AM actions to respond to changing climate by (1) structuring a framework to provide guidelines and recommendations for managers when applying genomics in AM, (2) summarizing genomic methods that could be applied in AM planning and monitoring, (3) reviewing current AM plant and animal examples that apply genomic techniques, and (4) highlighting a plant (big sagebrush *Artemisia tridentata*) and an animal (redband trout *Oncorhynchus mykiss gairdneri*) model system, where climate change has become a major threat and AM is likely to be an important management action. Thus, this study adds an essential, yet often absent, genomic component to the broader decision‐making AM framework, and further extends the application of AM from mostly plants to other organisms that may benefit from this conservation approach.

## 2 A FRAMEWORK OF APPLYING GENOMICS IN AM

**FIGURE 2:**
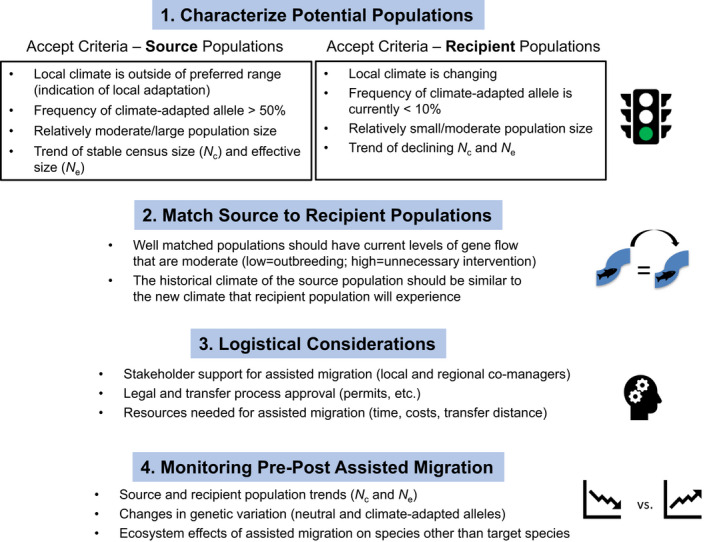
Flowchart for decision‐making process involved in applying genomics in assisted migration to natural populations

As various species from terrestrial and aquatic ecosystems face extirpation under ongoing climate change, conservation strategies will need to be implemented to avoid local extirpation or possible extinction of numerous taxa (e.g., Hoffmann et al., 2021). Developing conservation decisions is a complex process and case‐dependent, especially when relocation of individuals is required. Thus, a general framework is needed to aid the decision‐making process. Several studies have developed broad‐based frameworks on managed relocation applications, including AM, and emphasized the integration of scientific, social, legal, and ethical challenges (McLachlan et al., 2007; Schwartz et al., 2012; Vitt et al., 2010). With respect to the purpose of this paper, this section focuses on the scientific component and proposes four core concepts on applying genomics in AM: (1) characterize potential populations; (2) match source to recipient populations; (3) evaluate logistical details; and (4) monitor populations before and after AM (Figure 2).

The first component in the AM framework is to establish criteria for the characterization of potential source and recipient populations. There are differing perspectives on selecting preferred source populations for AM: one that maximizes overall genetic diversity and the other that focuses on climate‐adapted alleles to improve adaptive capacity in recipient populations. The perspective on maximizing diversity is that source and recipient matching attempts should carefully evaluate genetic variation in source populations to ensure the greatest adaptive capacity in AM‐established populations (Hoffmann et al., 2021); this adaptive capacity may aid AM‐established populations against novel stressors (Watson & Watson, 2015). Some researchers advocate using mixed‐stock sources to maximize genetic variation to enhance adaptive capacity (Burton & Burton, 2002), and others have shown that individuals with higher heterozygosity have higher probability of survival after translocation (Scott et al., 2020). However, selecting founding individuals solely to maximize represented diversity could reduce adaptive traits leading to outbreeding depression and phenotypes poorly suited to environmental factors (Edmands, 2007; Orr, 1996). For this reason, local adaptation and regional differentiation, such as distinct biogeographic units, are important considerations for source stock selection (Weeks et al., 2011) with a combination of selection strategies (Hoffmann et al., 2021).

Primary criteria for source populations for AM include evidence for local adaptation to climates outside of the preferred range (e.g., extreme heat or low precipitation), high frequency of climate‐adapted alleles (e.g., >50% frequency of alleles for high thermal tolerance; established through common gardens or genotyping candidate genes), relatively moderate to large population size to avoid excessive depletion, and trend data that indicate a stable census (*N*_c_) and effective (*N*_e_) size. Criteria for target recipient populations include local populations experiencing changes in climate that may lead to extirpation, frequency of climate‐adapted alleles that is currently low (e.g., <10%), relatively small‐to‐moderate population size, and trend data for declining *N*_c_ and *N*_e_.

Once potential populations have been characterized with criteria above, it is necessary to match source to recipient populations that would be most appropriate for translocations. In particular, well‐matched populations should have current levels of gene flow that are relatively modest since populations with low gene flow may be at risk of outbreeding depression when individuals are moved. In contrast, populations with existing levels of high gene flow are likely to naturally establish locally adapted alleles, and therefore, intervention is unnecessary. To facilitate the prediction of outbreeding depression, decision frameworks developed previously (e.g., Frankham et al., 2011; Hoffmann et al., 2021) can be used to include additional parameters of taxonomic status, selection intensity, genetic diversity, and isolation time. Further, the historical and current climate of the source population should be similar to the new climate that the recipient population is currently or predicted to experience. For example, a population of sagebrush that typically experiences a climate with large temperature extremes would be expected to be an appropriate match to source populations that have a similar climate or projected climate in the future (Richardson & Chaney, 2018; see the Web‐based mapping example in Section 5.1 for more details).

Third, there are often extensive logistical challenges involved when translocating individuals between source and recipient populations, even when they are well matched. Outreach to local and regional resource managers is needed to gain additional local perspectives that may be vital for successful outcomes. Support from resource managers is often key to developing final plans including legal approval, obtaining permits and public supports, and gathering necessary resources (e.g., time, money, equipment). Logistical hurdles may be particularly steep for AM outside of the current species range given concerns about impacts of introduced taxa on native species.

Finally, it is important to develop a comprehensive plan to monitor the biological consequences of AM, including genetics, in both source and recipient populations. This includes tracking population trends (*N*_c_ and *N*_e_), genetic variation of each population (neutral and candidate allele frequencies), and ecosystem effects to nontarget species in order to avoid negative and unintentional impacts. Each of these components may require extensive time and resources, but a thorough decision framework is needed to avoid loss of species necessary for ecosystem function.

## 3 GENOMIC METHODS FOR APPLICATIONS OF AM

There are two types of conservation management with different objectives: A common type is to maximize genetic diversity in restoration attempts to broadly account for genetic variation (e.g., Baums et al., 2019), and the other relatively rare type is targeting to move specific environmental‐ or climate‐adapted alleles with the potential for more beneficial outcomes. Applications of AM have rarely had the benefit of quantitative trait loci (QTL) markers to facilitate translocations of organisms, and thus have relied upon heritable phenotypic traits (quantitative genetics) or general estimates of genetic diversity based on putatively neutral markers. In plants, for example, seed transfer guidelines have largely been based on quantitative genetics from common garden studies in the absence of any type of genetic markers. While neutral genetic markers are widely studied and can be effective to evaluate population structure, and estimate existing levels of connectivity and genetic diversity of possible source and recipient populations, it provides limited information on adaptive traits related to performance. Examples in both aquatic and terrestrial organisms demonstrate how neutral markers have been used to infer demographic processes and reduce inbreeding depression (i.e., genetic rescue; Whiteley et al., 2015), but there are very few examples where candidate QTL markers for local adaptation have been applied for translocation efforts (e.g., Pacific lamprey, Hess et al., 2014; coral reefs, Schoepf et al., 2019) and especially AM to counter climate change.

**FIGURE 3:**
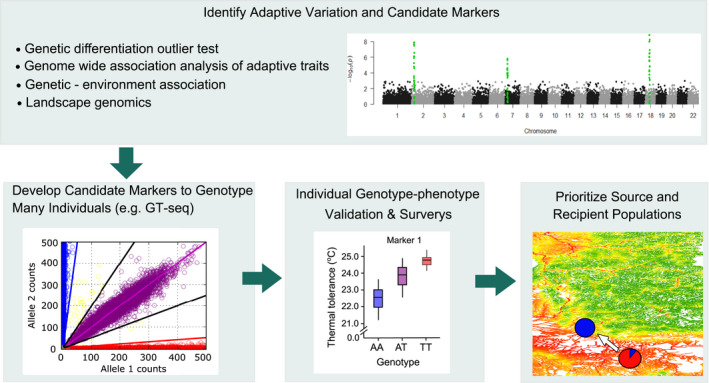
Schematic showing genomic approaches for applying assisted migration with adaptive alleles. The top panel illustrates a Manhattan plot with significant peaks for candidate loci from genome scan approaches, leading to development of specific candidate markers (e.g., genotyping‐thousands by sequencing; GT‐seq; Campbell et al., 2015) to test for genotype–phenotype validation in many individuals before considering intentional movement of adaptive alleles (red) from source to recipient populations

In order to effectively transfer climate‐adapted alleles from source to recipient populations, it is important to identify and characterize the genetic architecture of adaptive traits that improve performance and fitness of recipient populations that are experiencing climate change (Figure 3); but this approach has not yet been well utilized. While there are traits or phenotypes governed by only a few large effect genes, most adaptive traits have a complex genetic architecture and involve a large number of small effect genes (highly polygenic). Current genomic methodologies are most efficient for identifying genes/alleles with large effect. The reason is largely because sequencing technology has only recently advanced to the state where whole‐genome‐level variation can be reliably and cost‐effectively studied, and adaptive markers can be developed for screening natural populations. Reduced‐representation sequencing (RRS) approaches, for example, RAD‐seq (Baird et al., 2008; Bay et al., 2018; Elshire et al., 2011; Narum et al., 2013) and RNA‐seq (Bay et al., 2017), have recently been applied to detect signals of adaptive variation and applications for conservation management. However, RRS approaches are cost‐efficient only when a very small proportion of the genome is covered with relatively low marker density. Low marker density limits applications to identify adaptive variation in species with large genome sizes and genomic regions with low linkage disequilibrium (small haplotype or linkage blocks; Lowry et al., 2017; McKinney et al., 2017). More thorough genome coverage is possible with whole‐genome resequencing (WGR) and can be done cost‐efficiently when a reference genome assembly is available for the target species or closely related species (Fuentes‐Pardo & Ruzzante, 2017; Lou et al., 2021; Whibley et al., 2021). Further, efforts are underway to assemble genomes for vast numbers of species across the globe (Lewin et al., 2018; Rhie et al., 2021; Twyford, 2018), which should enable broad application of WGR.

Common garden experiments have been key in understanding trait variation in controlled experiments and identifying markers associated with phenotypic variation either through genome scans or targeted gene approaches. A detailed knowledge of adaptive traits and the underlying physiological mechanism is essential. Additionally, landscape genomics has been used to identify locally adapted genetic variation shaped by environmental factors in a wide variety of plants (e.g., Lasky et al., 2018), and terrestrial and aquatic animals (Grummer et al., 2019; Manel et al., 2010; Poelstra et al., 2014; Riginos et al., 2016). Once adaptive genetic variation is identified and verified, high‐throughput genotyping methods (e.g., amplicon sequencing, Campbell et al., 2015; DNA capture, Ali et al., 2016) can be applied to genotype large numbers of individuals and populations at relatively low cost (Meek & Larson, 2019). High‐throughput genotyping is a necessary step in AM because it can efficiently validate candidate markers in many populations, evaluate individual genotype–phenotype associations across highly heterogeneous landscapes at a large scale, improve the resolution of climate mapping models, and continuously monitor the status of populations so that source and recipient populations can be prioritized. However, it is necessary to validate candidate markers to adequately evaluate the potential benefits and risks before candidate markers are fully implemented for conservation applications including AM (Kardos & Shafer, 2018; Waples & Lindley, 2018).

These emerging techniques offer the potential to account for locally adapted alleles and phenotypic traits when considering source and recipient populations for AM. In particular, genomic vulnerability analysis estimates the adaptive capability and vulnerability based on geno‐environment modeling (Bay et al., 2018; Fitzpatrick & Keller, 2015; Layton et al., 2021), and therefore can be a powerful tool in evaluating populations for AM. In addition, the potential to increase the frequency of climate‐adapted alleles and diversity of life histories or phenotypic variation (i.e., “portfolio effect”) is a strong advantage to new techniques that will improve chances of successful application in natural populations. These new techniques also offer the potential to identify some of the major risks of AM including epistatic incompatible genes leading to outbreeding depression (Barmentlo et al., 2018) and identification of maladapted genes that are desirable to avoid transferring from source to recipient populations (Fitzpatrick & Reid, 2019; Hoffmann et al., 2021). However, we would like to raise a concern that biotic factors in source populations and potential biotic interactions in recipient populations are currently excluded in most investigations and should be considered whenever possible since they may contribute to selection pressure.

In summary, genomic techniques are becoming widely available to identify candidate climate‐adapted alleles and allow for effective characterization of allele frequencies in source and recipient populations (Aitken & Whitlock, 2013). Markers at the DNA level currently provide the strongest candidates to apply for AM, but gene expression and epigenetic biomarkers may prove useful if consistent signals can be verified for source and recipient populations.

## 4 SYSTEMATIC LITERATURE REVIEW OF GENOMIC AND EPIGENETIC APPLICATIONS IN ASSISTED MIGRATION

### 4.1 Methods for literature review

We employed the ISI Web of Science basic search engine to query the core collection database in a timespan ranging from 2000 to 2020 for the following terms: assisted migration, assisted evolution, assisted species migration, assisted colonization, assisted range expansion, assisted gene flow, and managed translocation. Each term was appended with the term “genetics” and “genomics,” respectively. Additionally, “seed zones” and “seed transfer guidelines” were queried to specifically search for plant‐related studies. The Social Sciences Citation Index (SSCI) and Arts & Humanities Citation Index (A&HCI) were excluded prior to search. Subsequently, the results were refined by excluding mismatching Web of Science categories. Search results were exported and saved as a table (Table S1). A detailed list of excluded categories can also be found in the table. After filtering for nonrelevant publication types (e.g., patents), categories (e.g., virology, chemistry, material sciences), and papers addressing AM in general, the remaining 729 publications were manually divided into groups of terrestrial plants (568), terrestrial animals (95), and aquatic animals (marine and freshwater) (66). These retained publications were further refined by the relevance to the focus of this review, which is the application of genetics/genomics in AM related to climate change. We classified papers into five categories: (1) not relevant—the papers show no evidence of moving individuals for climate change‐related management and no indication that genetic/genomic data could be used. Species distribution models with no genetic data were put in this category; (2) not relevant to AM for climate change as defined in this review but relevant to some other definitions of assisted migration or assisted colonization. Many of these papers related to efforts to restore genetic variation and avoid inbreeding depression caused by habitat fragmentation and population decline (i.e., genetic rescue); (3) relevant empirical paper but not applied. The authors conduct genetic/genomic analyses and mention results could be used to inform AM for climate change, but direct conservation actions have not occurred; (4) relevant empirical and applied. The authors use genetic/genomic analyses to inform AM for climate change and the movement is applied, and/or the authors are using genetic data to monitor after the movement; and (5) relevant review—review papers that discuss AM and the use of genetics or genomics to inform movement for climate change.

**FIGURE 4:**
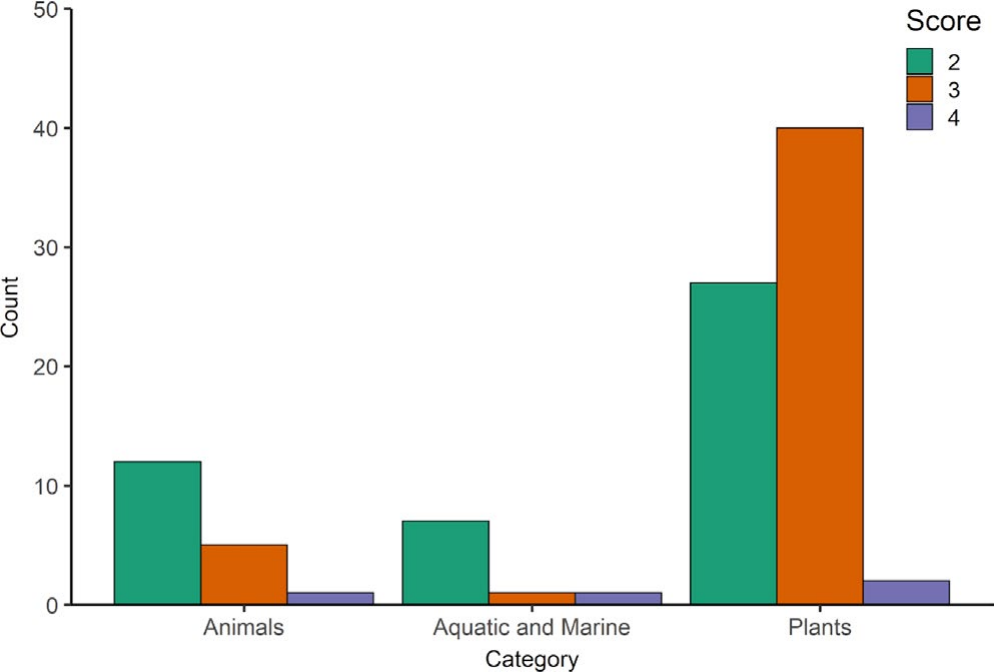
Counts of assisted migration (AM) papers in each scoring category for the three taxonomic groups. Scores 1 and 5, which represent “not relevant to genetics/genomics” and “review papers,” respectively, are not shown. Scores 2, not relevant to AM for climate change as defined in this review but relevant to some definitions of assisted migration or assisted colonization; 3, relevant empirical paper with genetics or genomics information but AM was not applied; and 4, relevant empirical papers and applied AM, are shown

Overall, the number of papers retained in the categories (3) or (4) was low and varied by system (Figure 4). The numbers of retained papers in these two categories were 42, 6, and 2 for plants, terrestrial animals, and aquatic animals, respectively. This highlights that there were roughly five times more plant papers than animal papers in the categories of interest.

### 4.2 Plants

In the face of climate change and habitat fragmentation, the immobility of the sporophyte and dependence on pollinators for some species create a unique set of plant vulnerabilities to the alteration in environmental conditions (Franks et al., 2014; Parmesan & Yohe, 2003). On the contrary, plants' sessile nature provides convenience in climatic adaptation studies, which often involve manipulating the environment to study corresponding plant responses (e.g., common gardens). Thus, a greater breadth of research can be found compared to the animal kingdom (Figure 4). While evidence for rates and types of plant responses to climate change are still not fully understood in many plant species, it has been shown that plants with diverse life histories adapt to changing environments by changing metabolism, phenology, or shifts in range distribution (Franks et al., 2014; Parmesan & Hanley, 2015; Richardson et al., 2017). However, due to geographical separation and limits to seed dispersal or habitat fragmentation, colonization to other suitable habitats might not be possible, particularly when species exhibit a long generation time (Aitken et al., 2008; Jump & Penuelas, 2005; Moran, 2020).

Foresters have been practicing AM in the form of assisted gene flow to introduce adaptive alleles long before the term was coined. The first seed transfer zones in North America were developed for Douglas‐fir (*Psuedotsuga menziesii*) from a heredity study started in 1912. Variable growth observed from different populations spurred the US Forest Service to create general seed transfer guidelines in 1939 and specific seed transfer zones for Douglas‐fir in 1942 (St. Clair et al., 2020). These guidelines were established prior to the broader recognition of climate change. Today, most commercially important tree species (e.g., Rehfeldt et al., 2014), many ecologically important shrubs (Richardson & Chaney, 2018), grasses (St. Clair et al., 2013), and forbs (Johnson et al., 2013) have published climate‐based seed transfer guidelines. These guidelines follow similar methodologies of assessing adaptive trait variation (e.g., growth, survival, and phenology) in common gardens: Regression models are developed using climatic predictors to explain the trait variability (e.g., Rehfeldt et al., 1999). The statistical models provide the capability to map the climate‐based clinal variation across the range of species and predict changes to trait variation based on current climate or future scenarios. Land management agencies in the United States and elsewhere use these mapped seed transfer zones to make decisions on developing seed sources for vegetative restoration and optimizing tree growth.

Early molecular approaches were limited in their ability to generate large numbers of variable genetic markers. These early studies focused primarily on how neutral genetic variations were affected by demographic changes. For plants in particular, these studies highlighted how past demographic changes influenced population genetic structure. For example, many studies have investigated the effects of past glaciations on genetic diversity and structure (e.g., Petit, 2003). These climatic events have strongly influenced the suitable habitats (i.e., glacial refugia) of many temperate and boreal species, and thereby affected the distribution of genetic diversity among populations. While these studies do not detect adaptive genetic variation that is essential for AM, they do provide valuable information by indicating how genetic diversity is arrayed across the landscape and illustrate how past climatic events have shaped populations of a species. For some species, these data can be used to infer corridors of gene flow, which can help inform AM and restoration efforts (Bocanegra‐González et al., 2018; Conroy et al., 2019; Xiao et al., 2015).

Another important source of genomic variation that can profoundly affect AM strategies is genome duplication (i.e., polyploidy). Polyploidy is prevalent in plants and can be achieved by means of genome duplication of the same species (autopolyploid) or different species (allopolyploid). Our review found only a few studies (Butterfield & Wood, 2015; Johnson & Vance‐Borland, 2016; Severns et al., 2013) that specifically consider cytotype (i.e., ploidy level) in addressing AM. However, a large body of literature shows that polyploidy affects species adaptation, gene flow, and niche preference (Blonder et al., 2020; Ramsey & Schemske, 2002; Schmickl & Yant, 2021), suggesting such data are critical in considering AM strategies. These strategies will likely differ considerably depending on the species. For example, quaking aspen (*Populus tremuloides*) harbors diploid and triploid clones. Triploids have been shown to have greater carbon assimilation than diploids, but potentially greater vulnerability to drought (Greer et al., 2018). In contrast, big sagebrush commonly harbors diploid and tetraploid plants where tetraploids have slower growth rates and seed production (Richardson et al., 2021), but greater uptake from soil surface water (Zaiats et al., 2020). Tetraploids are most prevalent in shallow soils and the most arid regions of the species range (Mahalovich & McArthur, 2004; Still & Richardson, 2015). This species is further complicated by widespread hybridization among subspecies, which generates varied phenotypes in ecotones (Richardson et al., 2012). The varied effects of subspecies, polyploidy, and hybridization over a large geographical distribution can make AM decisions complex.

**FIGURE 5:**
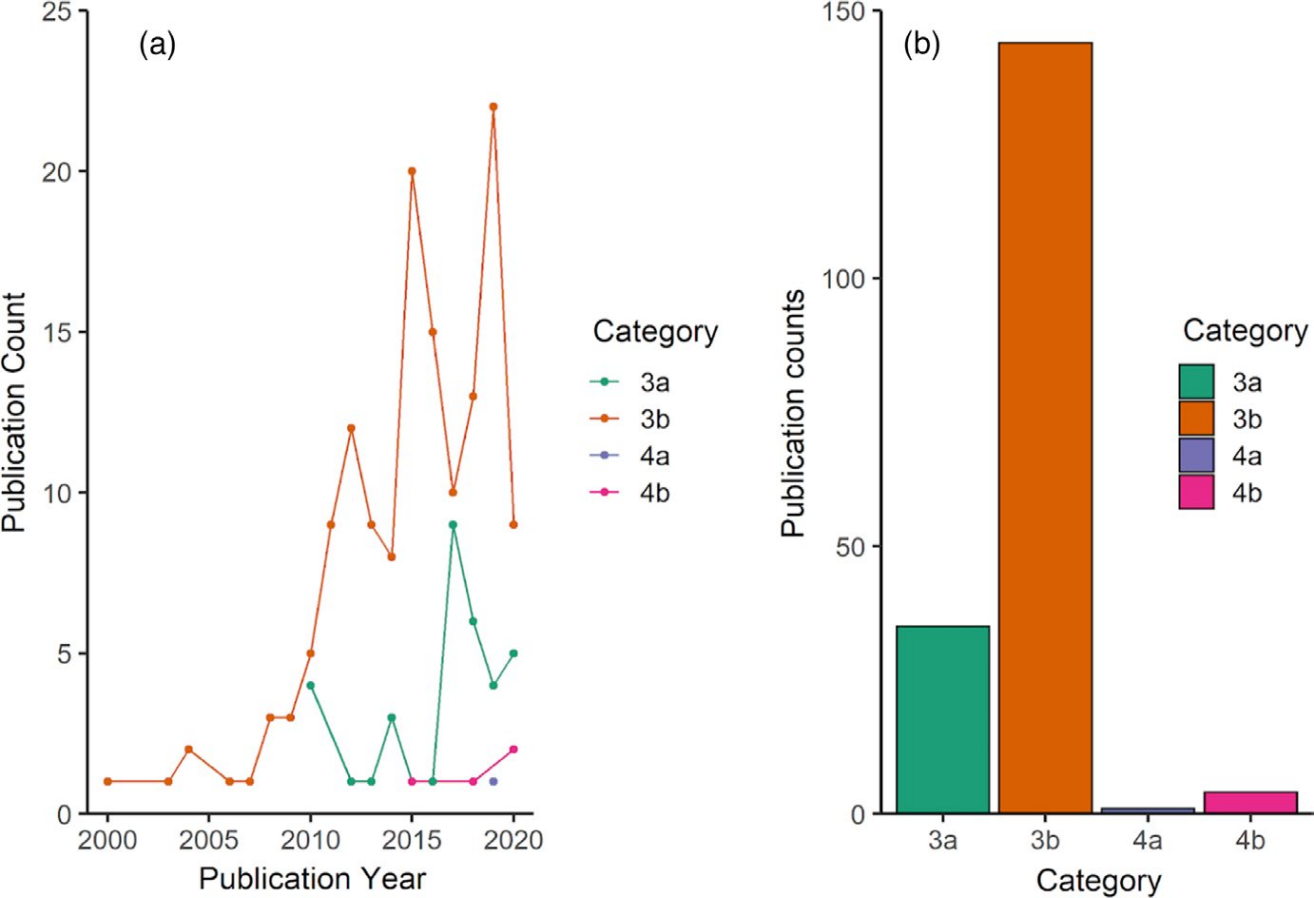
Number of papers in terrestrial plants based on the relevance to assisted migration for climate change over time (a) and total count of papers for each category (b). Papers were classified to category (3) if relevant empirical paper but not applied and (4) if relevant empirical and applied. Letters “a” and “b” represent papers using genomic‐based and trait‐based approaches, respectively

In the last two decades, a large number of quantitative and molecular studies have been performed to identify variables of local adaption and inform models to enhance the predictive performance of matching species to future climatic conditions. While the literature search shows genomic‐based approaches are currently the minority, these studies have been increasing over the last decade (Figure 5a). Trait‐based ecological genetic approaches comprise ~80% of plant studies that were identified as addressing AM (148 of 184 publications; Figure 5b). It is also important to note that often these trait‐based approaches provide the foundation to assess genomic‐based association (Browne et al., 2019; Csilléry et al., 2020; MacLachlan et al., 2018; Mahony et al., 2020). Of the AM plant literature that uses genomic approaches, eight publications use either association or QTLs, where three use an association solely with environmental variables. Throughout all studies, we find that multidisciplinary approaches, particularly when linking phenotypic with genotypic traits, are powerful to disentangle complicated relationships. Because of the varied life histories of plants, each study system will need to be specifically tailored. Results from our literature search find a variety of different reasons for AM, ranging from maintenance and conservation of genetic diversity to selection of sources for breeding programs. It has been shown that the majority of studies are linked to the economic importance of the study system, mainly trees, as opposed to efforts for conservation (Csilléry et al., 2020; MacLachlan et al., 2018; Milesi et al., 2019).

### 4.3 Freshwater and marine animals

Aquatic systems have suffered devastating consequences of global climate change. Increasing water temperatures plague both freshwater and marine ecosystems (Hoegh‐Guldberg & Bruno, 2010; Poff et al., 2002). Lakes suffer prolonged stratification (Kraemer et al., 2015), and streams experience continually decreasing discharge, leading to habitat loss or fragmentation (Leppi et al., 2012). Ocean acidification due to increased CO_2_ input has devastated major reefs around the globe (Hughes et al., 2019) and negatively impacted many other marine species. With substantial impacts to aquatic ecosystems, conservation action is necessary to ensure persistence of many species since natural mitigation of climate change effects is limited to altering distribution through movement or physiological adaptation.

Current genomic‐informed AM literature relating to aquatic systems focuses solely on reef‐building corals, which are foundation (habitat‐forming) species responsible for creating vast ecosystems that host immense biodiversity in ocean systems (Vergés et al., 2019). Therefore, the current literature for coral reefs provides the most useful guidance for developing strategies for AM that could be applied to other aquatic species and is covered in detail. A primary goal for maximizing the adaptive potential of AM corals is to maximize the genotypic diversity of translocated and outplanted corals. Rinkevich (2019) developed a "toolbox" of methods for restoring and maintaining sustainable coral reefs. In addition to assisted evolution, this toolbox includes assisted microbiome and epigenetic applications to ensure AM success in corals. Baums et al. (2019) provide a more detailed description than other researchers on how genomic methods could be implemented to achieve successful AM and suggested that genetic analysis must be performed to differentiate unique multilocus genotypes (MLGs) to determine coral genotypic diversity.

Due to increased ocean temperatures and acidification, coral bleaching has decimated these biodiverse ecosystems, and researchers are currently testing molecular methods for successful AM implementation efforts (Baker et al., 2004; van Oppen et al., 2014). Scientists are experimenting with assisted evolution through coral farming, where species are collected and propagated based on populations with higher tolerance to thermal pressure (Mascarelli, 2014; van Oppen et al., 2014; Vergés et al., 2019). Some researchers have developed frameworks that organize AM via direct translocation and outplanting of propagated coral species from viable source populations. For example, van Oppen et al. (2014) suggest using assisted evolution by hybridizing parents from multiple source populations possessing a combination of thermal‐adaptive traits and genetically modifying corals using CRISPR–Cas9 genome editing to restore diminishing coral populations throughout their original distribution range.

Considerations for AM in coral reefs have added complexity to account for their life cycle and symbionts. Most coral species are host to—and rely upon—symbiont algal species (*Symbiodiniaceae* spp.), and reef restoration efforts synchronously target these symbionts to ensure AM source population viability. Thermal adaptation of algal symbionts is a crucial element to the success of AM in coral species (Gates & Ainsworth, 2011; van Oppen et al., 2009), and tracking this biodiversity is recommended, although currently restricted by available genetic tools. In addition, phenotypic monitoring after AM implementation should target performance of the following phenotypes: bleach and disease resistance, high fecundity from sexual reproduction, high healing rate (as this adds to disease resistance), and high skeletal growth rate (Baums et al., 2019). While this monitoring can be time‐consuming, if beneficial phenotypes are observed in multiple genets within a population, subsequent genotyping for candidate genes and markers can increase future AM success.

A framework for successful AM in the reef‐building coral species *Acropora hyacinthus* has been developed by modeling a series of simulations to predict population persistence in response to increasing ocean temperatures (Bay et al., 2017) and offers a strategy that may be effective for other aquatic species. They used RNA sequencing data to analyze 20,883 SNPs for thermal‐adaptive association in closed populations of *A*. *hyacinthus* from two islands in American Samoa with differing temperature regimes. Simulation of ocean warming conditions and population genetics estimated potential evolution at thermally adaptive loci among the populations. The simulations predicted that annually introducing ten heat‐tolerant individuals resulted in the persistence of recipient heat‐intolerant populations instead of extinction if absent of AM. Models also identified a positive correlation between the number of thermal‐adaptive loci and extinction.

Although genomics‐informed AM of climate‐adapted alleles in freshwater species was nearly absent in our literature search, extending these methods to freshwater organisms will be an essential step in enhancing AM research under scenarios of climate change. While translocation has occurred for several aquatic species at broad and regional geographical scales, there were no direct applications that met our search criteria for genomics‐informed AM. Future applications of AM in freshwater systems could restore and rescue some of the most imperiled aquatic species, which are constrained to areas as small as headwater stream channels that are isolated from other populations.

### 4.4 Terrestrial animals

Conservation translocations or the deliberate movement of wildlife for the purposes of conservation are common in terrestrial animals (Batson et al., 2015; Seddon, 2010). A 2016 literature review of translocations among North American animals from 1974 to 2013 found 224 different vertebrate and invertebrate animal species had been translocated with birds and mammals being moved the most frequently (32% and 26%, respectively; Brichieri‐Colombi & Moehrenschlager, 2016). However, application of AM to address climate change concerns was much less common (only in four species). Many review papers on conservation translocations and AM of terrestrial animals highlighted the value of genetic information in conservation planning (Batson et al., 2015; Brichieri‐Colombi & Moehrenschlager, 2016; Flanagan et al., 2018; Kelly & Phillips, 2019; Seddon, 2010; Stralberg et al., 2019), but our literature review revealed that applications of genomic techniques to AM in terrestrial animal systems are rare. Most of the published literature presents a strategic framework for future AM efforts without providing examples or applications. Important recommendations from these reviews included (1) the importance of matching source and recipient populations, (2) value of sources and individuals with high genetic diversity, and (3) the potential for genomic techniques to aid in source population selection through enhanced understanding of population genetic variation and identification of gene–functional and gene–environment trait associations (Kekkonen & Brommer, 2015; Stralberg et al., 2019).

One example of environmental mismatch between source and recipient populations was the translocations of bighorn sheep (*Ovis canadensis*) in western North America (Malaney et al., 2015). This study used genetics (microsatellite loci and mitochondrial DNA) and environmental association analyses to investigate the effects of previous translocations on re‐established herds in ecologically disparate regions. Results showed translocated populations inhabit areas that are significantly different than their original habitat, which may have forced animals to use phenotypic plasticity to adjust to new condition and may make animals susceptible to new stressors. This emphasizes the importance of matching ecotypes to local conditions prior to translocation to ensure the success of AM populations. While Malaney et al. (2015) did not implement genome‐wide analysis, this study illustrates how applying genomic techniques in terrestrial systems could enhance screening of source populations. In contrast, Scott et al. (2020) found that individual heterozygosity of desert tortoises (*Gopherus agassizii*) estimated from a RAD‐seq genomic dataset was a strong predictor of translocation survival while translocation distance and geographical region of origin were not important predictors.

Common characteristics of taxa forwarded as candidate for AM in terrestrial systems include those experiencing local climate change, restricted range, patchy distribution, limited dispersal ability, small population sizes, and/or slow population growth. Many terrestrial AM candidates exhibit thermal sensitivity and are typically species of conservation concern. Specific AM frameworks bolstered by the use of genomic techniques have been proposed for the American pika (*Ochotona princeps*) and the tuatara (*Sphenodon punctatus*). The American pika is an alpine habitat specialist that exhibits hallmark features of AM candidacy including limited dispersal ability, slow population growth, restricted range, and genomic evidence of adaptive differences associated with environment (Henry & Russello, 2013). A detailed framework was presented for American pica AM by documenting habitat, climatic requirements, and physiological constraints, along with establishing translocation protocols and the use of genomic techniques to identify gene–functional trait associations. Further, the framework suggested enhanced evaluation of potential AM source populations. Additional genomic work to identify adaptive loci has been conducted in this species and a Himalayan pika (*Ochotona roylei*), which will provide valuable information for future AM and other conservation efforts in pikas (Lemay et al., 2013; Solari & Hadly, 2018; Solari et al., 2018; Waterhouse et al., 2018).

Other examples of applied AM include ectothermic species of concern due to changes in climate. First is in the tuatara, an endangered reptile endemic to New Zealand that is considered highly vulnerable to extinction under climate change scenarios due to its restricted distribution, small population sizes (Gaze, 2001), and temperature‐dependent sex determination (Cree et al., 1992). Under proposed framework (Miller et al., 2012), AM for the tuatara would be established as a set of conservation experiments featuring comparisons between wild and AM‐established populations. Another study also suggests that AM efforts be established as conservation experiments (Watson & Watson, 2015), but advocate AM for common species as a proactive conservation effort. Finally, AM has also been suggested as a potential strategy to ensure the persistence of the Quino checkerspot butterfly (*Euphydryas editha quino*; Parmesan et al., 2015), which is undergoing a climate change‐induced range shift.

In summary, multiple sources consider AM a viable future conservation strategy for a diverse set of range and dispersal‐limited taxa undergoing climate‐induced declines in terrestrial systems. Applications of genomic techniques to AM would be expected to improve pairing between source and recipient populations and likely improve the potential for successful outcomes for translocated taxa.

### 4.5 Commonalities and differences

Across plants and animals, a number of consistent themes emerged, in particular from the review papers and proposed AM frameworks. In general, the number of papers using genomics to inform AM is low across systems (Figure 4). As discussed above, both plant and animal AM plans would benefit from identifying and moving adaptive loci related to the environmental stressor of concern, such as thermal tolerance. The identification of these loci may then guide the selection of source individuals to move to recipient populations. Besides adaptive loci, researchers often discuss other important metrics from neutral loci. Although population structure and connectivity may be estimated from genetic methods and applied to AM planning, the increased precision and power from genomic data may improve AM outcomes. In addition, papers from both plant and animal literature present the idea that AM best practices should include multiple metrics and not strictly metrics derived from genomic data. It is imperative to also develop an understanding of source and target population habitats, population sizes, climatic vulnerabilities, and other metrics not determined by genomics. The similarities in overall frameworks presented in existing reviews of AM, including the incorporation of genomic data and inferences, mean that researchers across taxonomic groups are already trying to address similar challenges and questions in similar ways.

Differences have occurred in the timescale and methods between plants and animals. As previously described, plants have a longer history of studies providing inferences and conclusions, which could also be made through the use of genomic techniques by using techniques with a long history. The use of seed zones provided a methodology to assess questions related to AM without the emergence of genomic techniques, and without similar methods in animals. The closest application would be the general approach of choosing source populations from similar populations, either geographically or ecologically. These guidelines have also been more broadly discussed with relation to translocation than AM. Although these ideas have consistent use in the animal literature over the past two decades, AM is a much more recent endeavor. Besides seed zones, the second major difference observed in reviewing literature between plants and animals comes from the use of trait‐based metrics in plant studies. For an array of reasons, such as study logistics, QTL and other trait‐based methodologies are much less common in animals than plants. These differences highlight that AM outcomes may be improved by increased collaboration and discussion across taxonomic groups.

## 5 CASE STUDIES

Considering there are a limited number of examples that have applied genomics in AM under climate change, especially in terrestrial and aquatic animals, we provide two case studies using the framework for AM, to encourage broader range of target species for future research. These two case studies, one in a long‐lived plant species and one in broadly distributed freshwater fish, are examples of what is known from recent genomic work and the potential process for applying AM to improve adaptive capacity under scenarios of climate change.

### 5.1 Sagebrush

Big sagebrush is a cornerstone of cold desert ecosystems in western North America. This species is critical in mitigating soil erosion, fostering plant and animal biodiversity, storing carbon, and providing cover and forage for wildlife. The large ecological breadth of big sagebrush lends itself to subspecific divergence. This species has three predominant subspecies that occupy distinct niches in the sagebrush biome. Basin big sagebrush (*Artemisia tridentata* subsp. *tridentata*) and Wyoming big sagebrush (*A*. *tridentata* subsp. *wyomingensis*) occupy lower elevations and can typically have overlapping distributions depending on the soil moisture regimes. Mountain big sagebrush (*A*. *tridentata* subsp. *vaseyana*) occupies montane ecosystems at higher elevations. Loss of sagebrush ecosystems has been immense since the pre‐Columbian era, with one‐third to one‐half of this ecosystem being lost (West, 2000). While earlier 20th‐century losses of these ecosystems came from removal for agriculture, current losses are principally caused by the interaction between cheatgrass (*Bromus tectorum*) and increasing wildfire frequency leading to reductions in biodiversity and soil stability (Germino et al., 2016). The warm‐dry regions of the sagebrush ecosystem, primarily occupied by Wyoming big sagebrush, are particularly susceptible to the cheatgrass–wildfire cycle and decrease resilience and resistance to these disturbances (Chambers et al., 2014).

AM strategies will need to target areas of the sagebrush ecosystem that is stable or will expand in this century. This undertaking requires an understanding of the interactions between cheatgrass, wildfire, and climate change. Studies have shown spatial correspondence between the predicted mid‐century (2050s) range contraction of Wyoming big sagebrush climate niche model and higher cheatgrass cover (Bradley et al., 2018; Still & Richardson, 2015), supporting the observation that sagebrush resistance and resilience decline in warm‐dry environments (Chambers et al., 2014). Climate niche models provide a good starting point for planning source and target geographical locations for AM. As with any model, there are a number of assumptions with niche models. Incorporating other important factors (e.g., plant competition) will be important in refining our climate predictions, especially at finer spatial scales.

An essential component for AM is discerning intraspecific adaptive genetic variation. Movement of seed sources outside of their adaptive niche can lead to maladaptation and restoration failure. Common garden studies provide an effective research approach to elucidating adaptive variation in plants. Big sagebrush common gardens have shown adaptive variation in cold hardiness, phenology, growth, and seed yield (Chaney et al., 2017; Richardson et al., 2017, 2021). These trait responses are primarily shaped by climate. For example, cold temperatures predict patterns of mortality with populations from continental climates able to survive these conditions better than populations from warmer, western regions of the range (Chaney et al., 2017). The patterns of mortality are related to different physiological strategies in freeze resistance during early spring (Lazarus et al., 2019). The linear functions that describe these trait responses can be mapped and compiled to develop seed transfer limits (Richardson & Chaney, 2018). Since these functions are based on climatic predictors, developing a prediction for climate change is simply exchanging contemporary climate values for those that have been adjusted using ensemble climate change scenarios.

**FIGURE 6:**
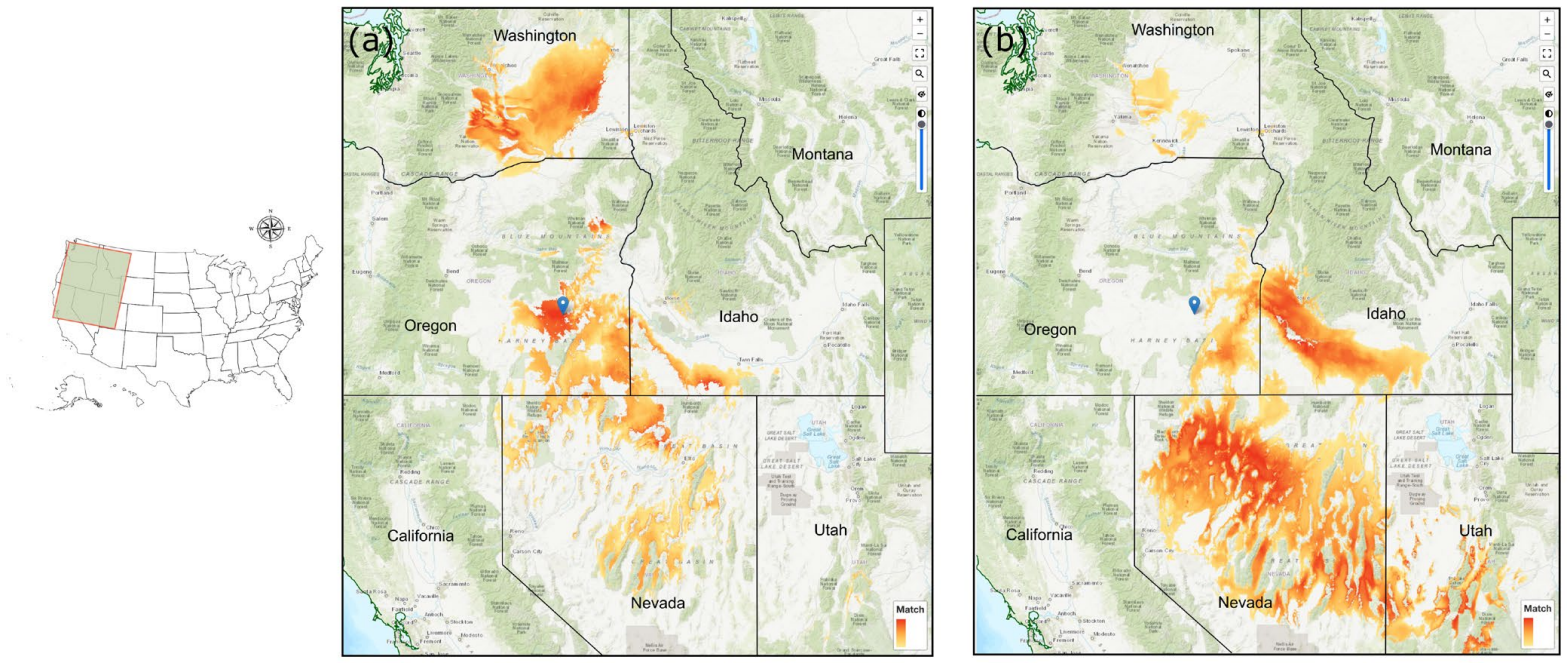
Seed transfer distances for Wyoming big sagebrush for a site in central Oregon (blue pin) created in the CSRT. (a) shows contemporary climate seed sources suitable for the site; (b) shows mid‐21st‐century climate seed sources. The color gradient indicates climate similarity to the chosen site. Note that by mid‐21st century, adapted seed has shifted to warmer elevations and latitudes east in the Snake River Plain and south in the Great Basin

Developing mapping tools that integrate species/subspecies climatic niche models, population‐level adaptive variation, and climate change models based on global climate models (GCMs) are needed for the implementation of AM. A Web‐based mapping platform, the Climate Smart Restoration Tool (CSRT, https://climaterestorationtool.org/csrt/), is being developed for big sagebrush and other species that utilize common garden and niche model data. The CSRT requires only geographical coordinates, subspecies designation, and a climate scenario from the user to map seed transfer limits for the specific site. The tool uses cold hardiness and phenology trait models to determine transfer limits. Subspecies niche models are used to constrain mapped projections to within the predicted range. An example of the CSRT is provided in Figure 6, most extant big sagebrush populations will be climatically maladapted to mid‐21st‐century climates. Therefore, AM is needed to maintain resilient stands of this species.

Development of genomic tools to address AM in big sagebrush has trailed behind quantitative research, as has been common for many plant species. Genomic techniques can augment our understanding of sagebrush quantitative traits in several ways. First, for many plant species the taxonomic position and species boundaries are not completely understood. Genomics can be used prior to or during a common garden study to delineate relationships and cytotypes among populations and provide valuable inference into assessing quantitative traits (Gompert & Mock, 2017). For example, amplicon sequencing was used to help infer subspecies of *A*. *tridentata* common garden studies (Richardson et al., 2012). These data helped group populations into their respective subspecies for quantitative trait analyses. Second, neutral landscape genomics can infer demographic parameters such as effective population size and connectivity. Population genetic structure or demographic models can be used in coordination with seed transfer zones and added as an additional constraint (Massatti et al., 2020). Third, genomics provides an opportunity to infer the impact of selection on a wider array of evolutionary pressures compared with quantitative traits that are principally focused on climate. For example, herbivore browsing selection could be deduced from genomic approaches by identifying the genes involved with chemical defenses and their interactions with herbivores.

### 5.2 Redband trout

**FIGURE 7:**
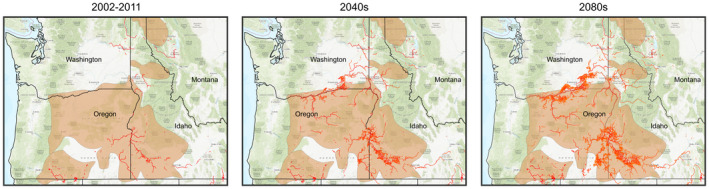
Predicted impact of global warming on redband trout habitats. Predictive model based on global climate change A1B warming trajectory (IPCC, 2007) predicts that redband trout habitat (shaded area) will have more streams with August mean stream temperatures exceeding 20°C (streams highlighted in red) in the 2040s (2030–2059) and the 2080s (2070–2099) (Isaak et al., 2017). Desert redband trout populations located in the Western Snake River Plain will likely be significantly impacted

Redband trout (*Oncorhynchus mykiss* ssp. *gairdneri*) is a subspecies of *O*. *mykiss* inhabiting interior regions of western North America as compared to coastal‐origin rainbow trout (Figure 7). Habitat characteristics of redband trout populations differ significantly in elevation, shading, flow, substrate, and thermal regime (Meyer et al., 2010), resulting in local adaptation to a spectrum of conditions (Narum et al., 2010, 2013). While redband trout occupy a wide range of habitats, their distribution has declined rapidly as a result of anthropogenic influences such as habitat alteration, migratory barriers, and changing climate (Muhlfeld et al., 2015). Loss of redband trout could cause major disruptions in local ecosystems since they are typically the primary fish species in small, isolated creeks throughout the range. While climate change is predicted to be the most prominent factor affecting the geographical distribution of this species (Figure 7), some redband trout in the high desert basins of Oregon, Idaho, and Nevada have successfully adapted to harsh conditions where summer water temperatures are close to their upper thermal maximum (~29°C), but the opportunity for further adaptation is likely restricted (Behnke, 1992; Chen et al., 2018; Rodnick et al., 2004). Also, natural migration and gene flow have been limited by anthropogenic physical barriers (e.g., water diversions and dams). For these reasons, AM might be an appropriate strategy to consider in redband trout conservation along with accumulating genomic knowledge and tools for this species.

Much past ecological and evolutionary research in redband trout conservation has the potential to be applied to AM in this species. However, the most promising area of applying genomics in AM for redband trout is the knowledge on the genomic basis of local adaptation and particularly the climate‐related adaptive traits. Redband trout currently inhabit some of the warmest habitat of any salmonid, and therefore, it has been used as a “winner” model to study mechanisms of thermal adaptation (Narum & Campbell, 2015; Narum et al., 2010). Underlying adaptive physiological traits and genes have been identified for thermal tolerance, energetics, and cardiorespiratory functions (Chen et al., 2018; Chen, Farrell, Matala, & Narum, 2018). Most recently, the *CERK* (ceramide kinase) gene has been shown to be associated with upper thermal tolerance and cardiac function (Chen & Narum, 2021). If validated, candidate genes from these studies can be applied to genetic vulnerability analysis models to screen source populations/individuals and identify recipient populations after evaluating their adaptive potential to climate change.

Within redband trout, there are several major phylogenetic divisions with many genetically distinct groups (Currens et al., 2009) that need to be considered when performing AM to preserve genetic diversity and reduce genetic risk. One major biotic threat to native redband trout populations is the introgressive hybridization with nonnative salmonid species (e.g., cutthroat and coastal‐origin rainbow trout) (Kozfkay et al., 2011; Muhlfeld et al., 2015; Wishard et al., 1984). Therefore, by introducing adaptive alleles from populations of the same species, AM might provide the added value of preserving “pure” populations in addition to the intended purpose of addressing climate change scenarios. Nevertheless, performing AM outside the native distribution of redband trout raises concerns about invasion into new habitats and would require consideration of other effects such as potential hybridization with endemic species in the expanded range.

Redband trout are ancestral tetraploids owning to the salmonid‐specific whole‐genome duplication event occurred about 100 million years ago. It is estimated 50% of protein coding genes and all miRNA genes of the *O*. *mykiss* genome still retain duplicated copies (Berthelot et al., 2014). More gene copies and an increased amount of genetic variation likely contributed to the successful evolution of redband trout across their wide geographical distribution (Allendorf & Thorgaard, 1984). Results of the successful evolution are many small demes, complex population structure, rapid response to selection, and genomic divergence. Although polyploids in general may be less susceptible to inbreeding and outbreeding depression by virtue of having more allele copies, applying AM in redband trout should follow the same decision process in Section 2, especially as much of the genome has undergone rediploidization (Pearse et al., 2019).

## 6 CONCLUSIONS

Rapid climate change has been and will continue to be a major threat to global biodiversity in the 21st century. We anticipate that AM can be a useful conservation option and should be considered for species/populations currently under climate‐induced risk, especially for those that are less mobile (e.g., blocked by migratory barriers) or less adaptable (e.g., low standing genetic diversity). The feasibility of AM involves vulnerability assessment, abiotic and/or biotic interaction in recipient ecosystems, and likelihood of long‐term success. Answering these questions is not an easy task as they are often case‐dependent and require empirical research at regional scales but may be beneficial for long‐term conservation of biodiversity on earth.

Recent genomic research in non‐model species across the globe has begun to reveal mechanisms related to environmental adaptation. Such knowledge is expected to contribute toward applications of AM under scenarios of climate change, but current use of genomic tools for this purpose has been limited. Incorporating high‐quality genomic data can facilitate AM by selecting the most suitable source individuals for recipient populations that are currently struggling with changes in climate. Although AM strategies will be case‐dependent, initial models of success can provide reference for the broader community and this review summarizes initial efforts in AM in plant and animal species along with insights toward applying this tool for conservation management. Conservation requires collaboration, and successful AM is expected to involve a large team effort outside the scientific community including outreach to the public and critical policymakers and resource managers.

## CONFLICT OF INTEREST

The authors declare no conflict of interest.

## Supporting information

Table S1

## Data Availability

No new data were generated for this article. A list of references returned from the literature search on existing studies are summarized and saved in the supplementary spreadsheet.
